# Atomistic Study of Interactions between Intrinsic Kink Defects and Dislocations in Twin Boundaries of Nanotwinned Copper during Nanoindentation

**DOI:** 10.3390/nano10020221

**Published:** 2020-01-28

**Authors:** Xiaowen Hu, Yushan Ni, Zhongli Zhang

**Affiliations:** 1 Department of Aeronautics and Astronautics, Fudan University, Shanghai 200433, China; xwhu16@fudan.edu.cn; 2 Shanghai Institute of Measurement and Testing Technology, Shanghai 201203, China; zhangzl@simt.com.cn

**Keywords:** defective twin boundary, kink-like defect, dislocations-defect interactions, surface accumulation, dislocation transmission, nanoindentation, twinning partial slip

## Abstract

In order to study the effects of kink-like defects in twin boundaries on deformation mechanisms and interaction between dislocations and defects in twin boundaries under localized load, nanotwinned Cu with two defective twin (TDT) boundaries is compared with the nanotwinned Cu with two perfect twin (TPT) boundaries, and nanotwinned Cu with single defective twin (SDT) boundary and single perfect twin boundary by simulating spherical nanoindentations using molecular mechanics. The indenter force-depth and hardness-contact strain responses were analyzed. Results show that the existence of intrinsic defects in twin boundary could reduce the critical load and critical hardness of nanotwinned material. A quantitative parameter was first proposed to evaluate the degree of surface atom accumulation around the indenter during nanoindentation, and it can be inferred that the surface morphology in TDT changes more frequently than the surface morphologies in TPT and SDT. The atomistic configurations of incipient plastic structures of three different models were also analyzed. We found that the intrinsic defects in twin boundary will affect the incipient plastic structures. The formation of twinning partial slip on the defective twin boundary happens before the contact of the dislocation and twin boundary. The kink-like defects could introduce Frank partial dislocation to the twin boundary during interaction between dislocation and twin boundary, which was not detected on the perfect twin boundary. In addition, the area of twinning partial slips on the upper twin boundary in the incipient plastic structures in SDT and TDT are larger than the twinning partial slip area in TPT, which results in the reduction of the critical hardness in SDT and TDT. The kink-like defects could also block the expansion of twinning partial slip on the twin boundary. Furthermore, we investigated the dislocation transmission processes in three different models. It is found that the dislocation transmission event could be delayed in model containing single defective twin boundary, while the transmission process could be advanced in model containing two consecutive defective twin boundaries. The quantitative analysis of dislocation length was also implemented. Result shows that the main emitted dislocation during nanoindentation is Shockley partial, and the dislocation nucleation in SDT and TDT is earlier than the dislocation nucleation in TPT due to the existence of defects. It is inferred that the intrinsic defects on twin boundaries could enhance the interaction between dislocations and twin boundaries, and could strongly change the structure evolution and promote the dislocation nucleation and emission. These findings about kink-like defects in twin boundaries show that the inherent kink-like defects play a crucial role in the deformation mechanisms and it should be taken into consideration in future investigations. Single defective twin boundary structure is recommended to delay the transmission and block the expansion of twin boundary migration. Some of the results are in good agreement with experiments.

## 1. Introduction

Recently, in order to simultaneously improve the mechanical properties and the thermal stability, a new strategy which is engineering the defects in metals rather than alloying has been suggested by Lu [1,2]. This sustainable “plain” approach provide an idea to tailor the materials by manipulating imperfections across different length-scales while do not change the chemical compositions. Some researchers have studied on various imperfections in crystalline materials. For example, Meyers et al. [3] study the growth of nanosized voids in ductile failure of metals by performing analytical and atomistic calculations for the tensile behavior of nanocrystalline copper. Lu et al. [4] discussed the role of nanoscale internal grain boundaries in strengthening and preserving ductility of materials. Among these imperfections in crystalline materials, coherent twin boundary (CTB), a kind of planer defect in nanocrystalline materials, has attracted extensive attention due to its low-energy and stability in high-temperature and radiation environment [5,6,7]. Researches show that twin boundaries are difficult to slide, and they act as barriers in the dislocation transfer events and resist the inclined dislocation slip [8,9]. Furthermore, face-centered cubic metals containing nanoscale CTBs have an excellent performance on the tensile strength and ductility [4,10]. The strength of nanotwinned copper increases with decreasing twin thickness and reaches a maximum at 15 nm [11]. Gradient structure in the nanotwinned metals will further enhance the strength of material [12]. Meanwhile, some researches focused on the mechanism of interaction between dislocation and twin boundary and the nucleation of dislocation at twin boundary have been reported. You et al. [13] classify the deformation mechanisms into three modes according to the slip directions of the dislocations nucleated on the twin boundary. Wu et al. [14] learned the reaction on twin boundary of 60° dislocation and Shockley partial. They concluded that the immobile dislocations at twin boundary lead to strain hardening, and mobile Shockley results in good ductility. Lu et al. [15] reported a transition of dislocation nucleation from twin boundaries to twin boundary/grain boundary junctions in nanotwinned copper, which happens at critical twin lamella spacing.

It is worth mentioning that Wang et al. [16] reported that as-grown coherent twin boundaries are inherently defective with large amount of kink-like defects (which are called defective twin boundaries, DTBs), and these defects play a crucial role during plastic deformation. Despite numerous previous studies on nanotwinned metal, the kink-like defects in twin boundaries have rarely been taken into account. Recently, only few researchers have studied the effects of these kink defects on the deformation mechanism. Fang et al. [17] learned that the kink defect in the twin boundary could change the interaction between dislocation and twin boundary from dislocation transmission to dislocation absorption. Xing et al. [18] performed the uniaxial tension on copper nanowire with kink-like defective twin boundaries and the kink density substantially affect the yielding mechanisms.

However, previous studies on the defects both employed uniform loads such as tension and shear. It is still unclear about the mechanisms related with intrinsic twin boundary defects under localized external load. The mechanisms of the interaction between kink defects or incoherent components in twin boundaries and threading dislocations still require further investigation, and new approaches should be applied to study the localized deformation. Nanoindentation is a significant method to obtain the material properties with highly localized stress, and it has been widely adopted to investigate the dislocation reaction on coherent twin boundaries with localized deformation. Bufford et al. reported [19] the work hardening capacity and high plasticity in highly twinned aluminum with incoherent twin boundaries during nanoindentation in a transmission electron microscope (TEM). Huang et al. [20] simulated the nanoindentation on nanotwinned Ta with various twin lamella thicknesses by using molecular dynamics, and analyzed the propagation of dislocations and migration of twin boundaries.

In order to study the interaction between dislocations and defects on the twin boundary under localized load, kink-like defects are introduced to twin boundaries of nanotwinned copper to perform nanoindentation simulations in this paper. The load and hardness responses of nanotwinned Cu with two perfect twin boundaries is compared with nanotwinned Cu with one defective twin boundary and one perfect twin boundary and nanotwinned Cu with two defective twin boundaries. The surface accumulation of three different models are also analyzed. In addition, the effects of kink defects are discussed by comparing the incipient plastic structures, twinning partial slip, dislocation transmission and interaction between twin boundaries through the atomic structures at different indentation stages. The changes of dislocation length during nanoindentation are also analyzed quantitatively.

## 2. Models and Methods

**Figure 1:**
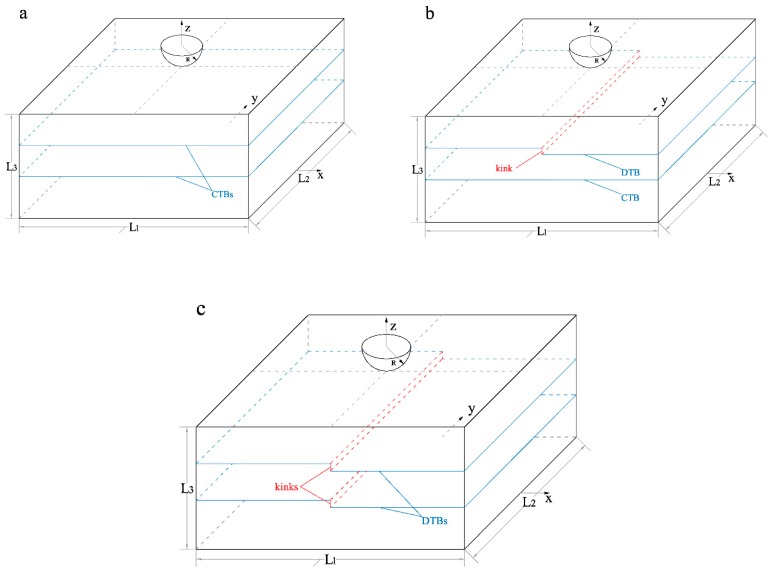
Schematic representation of nanoindentation with different models. (**a**) Two perfect twin (TPT) model, (**b**) single defective twin (SDT) model, and (**c**) two defective twin (TDT) model.

According to experimental observation [16], the kink-like defects inherently exist in the twin boundaries of nanotwinned Cu. In order to investigate the influence on material properties and deformation mechanisms of this kind of defect and compare the differences of deformation during nanoindentation between nt-Cu with or without defects, the three models with different twin boundary structures were used in this work. As illustrated in Figure 1, the models of our indentation simulations are constructed in a three dimensional Cartesian coordinate system, and all these models have the same sizes in three directions where L_1_ = 19.4 nm, L_2_ = 21.3 nm, L_3_ = 10.2 nm. Each model contains about 360,000 atoms, with the bottom atoms whose z coordinates are less than 0.78 nm fixed as the bottom boundary to avoid the overall rigid motion of the model. The midpoint of the bottom of each model is set as the origin of the coordinate system. Two twin boundaries are included in each model, which are placed at 3.13 nm and 6.89 nm below the surface separately. As shown in Figure 1a, both of the twin boundaries in the first model are perfect without defect. In Figure 1b, one kink-like line defect, which is $\Sigma 3\{11\bar{2}\}$ segment, is placed at the middle of the upper twin boundary and the height of the kink is about 3 atomic layers and the lower twin boundary is perfect. In Figure 1c, both of the twin boundaries have a kink-like line defect in the middle. These three models (according to their twin boundaries type) are named as nt-Cu with two perfect twin boundaries (TPT) model, nt-Cu with single defective twin boundary (SDT) model and nt-Cu with two defective twin boundaries (TDT) model, respectively. The specific selection of the defect position, direction and spacing is determined according to the overall size of the model, the maximum indentation depth in the simulation and the spacing of the twin boundaries. The purpose is to make the highly localized stress field in the nanoindentation process have sufficient interaction with the twin boundary defects in the initial yield stage. Periodical boundary conditions were applied in horizontal directions in all three models in order to minimize the boundary effect, while the surface boundary is free in the vertical direction. During the simulation, the plastic zone which contains all dislocations and other structures would not reach the bottom boundary, thus this set of material size is sufficient to investigate the structure of plastic deformation. The crystal directions of the top grain and bottom grain of three structures are set to be $\left[1\bar{1}0\right]$, $\left[11\bar{2}\right]$, and [111], and the crystal directions of the middle grain are set to be $\left[\bar{1}10\right]$, $\left[\bar{1}\bar{1}2\right]$, and [111]. All adjacent grains are symmetric with respect to the grain boundaries between them and the crystal direction of these boundaries is [111], such that the grain boundaries between the adjacent grains can be regarded as twin boundaries.

The embedded atom method (EAM) potential developed by J.B. Adams et al. [21] was chosen in our simulations using LAMMPS code [22]. This potential is widely adopted to be used in studying the twin dislocation interactions in molecular simulations [23,24,25]. The influence of temperature on plastic deformation and dislocation interactions is not considered in this work, microcanonical ensemble is taken during the simulation process and the temperature of thermostat atoms is controlled by rescaling the atoms velocities. The starting temperature is OK. Noting that a low temperature, even at OK, is helpful for analysis of deformation mechanism and is commonly adopted in MD nanoindentation simulation [26,27,28]. The spherical indenter used in this work is 4 nm in radius, and a repulsive potential field is used to describe the force on each atom in contact with the indenter:

(1) $$V\left(r\right)=\{\begin{array}{cc} k{\left(R-r\right)}^{3}, & r\leq R\\ 0, & r>R \end{array}$$

where *k*, the contact stiffness, has been set to $10eV{Å}^{-3}$, and *r* is the distance between atom and center of the indenter, *R* is the radius of the indenter. The time step is set as 1 fs, and the indenter moves downward with speed of 10 m/s. The maximum indentation depth is 30 Å. After simulations, the dislocation extraction algorithm (DXA) [29] and OVITO (Open Visualization Tool, a scientific data visualization and analysis software for working with molecular and other particle-based data) [30] were used to describe and identify the dislocations in the structures.

## 3. Results and Discussion

### 3.1. Force-Indenter Depth Responses

**Figure 2:**
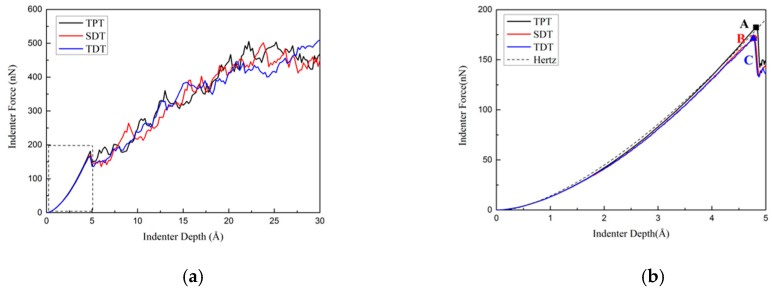
(**a**) Force-indenter depth responses of three different models during nanoindentation. (**b**) Partial enlargement of the elastic stage of the force-indenter depth responses, dash line fitted by Hertz theory.

In order to study the influence of defective twin boundaries on the critical load during nanoindentation and verify the correctness of our simulation, the force-indenter depth responses with the three models during nanoindentation are shown in Figure 2a. The vertical force on the indenter is calculated by summation of the z-direction force on the atoms in contact with the indenter. And the partial enlarged detail of the elastic stages during indentation are shown in Figure 2b.

According to Hertzian elastic contact theory [31], the relationship between contact force and indenter depth during elastic stage of spherical indentation could be expressed as:

(2) $$P=\frac{4}{3}E^{*}\sqrt{Rh^{3}}$$

where *R* and *h* are indenter radius and indenter depth. The reduced modulus $E^{*}$ is defined as

(3) $$E^{*}=\frac{E^{\prime }}{1-{\nu^{\prime }}^{2}}$$

where $E^{\prime }$ and $\nu^{\prime }$ are anisotropic Young’s modulus and anisotropic Poisson’s ratio of Cu in [111] crystal orientation. Applying experimental data and Voigt approximation [32,33], the result of calculation of reduced modulus is $E^{*}=211GPa$. The Hertz solution [31] was added in Figure 2b with dash line.

It can be seen that the curves at the elastic stages are fitted well with Hertzian contact theory curve. The force on the indenter increases with the increase of indenter depth during elastic stage, until the first drop of load occurs. The yielding points in TPT, SDT, TDT are labeled as A, B, and C in Figure 2b, respectively. All three models yield at the same indenter depth 4.8 Å, while the critical loads for TPT, SDT, and TDT are 182.22 nN, 171.74 nN, and 171.05 nN, respectively. This means the defects in the twin boundary will not lengthen or shorten the elastic stage of nanoindentation, but will reduce the critical indenter force of nanotwinned copper, which corresponds to the differences between the incipient plastic structures of the three models. After the critical load, many drops of loads happen in the subsequent deformation process in all three models. It is known that these sudden decreases in load responses indicate the changes of the inner structure, including dislocation nucleation and emission, twin boundary migration, and many other dislocation interactions mechanisms. These curves which contain many drops during the nanoindentation process are in good agreement with some experiment results [19,34]. We will discuss these mechanisms below by investigating the microstructures of dislocations.

### 3.2. Hardness-Contact Strain Responses

**Figure 3:**
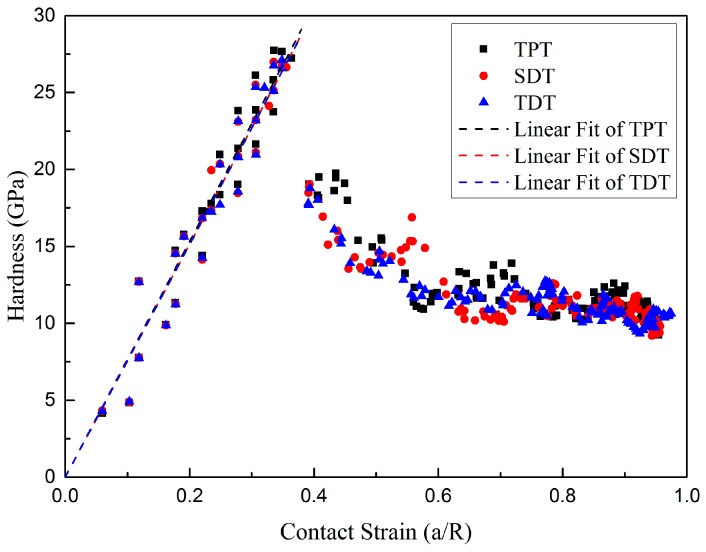
Hardness-contact responses of three different models during nanoindentation, linear fits of three various models during elastic stages.

The hardness-contact strain response is an effective way to evaluate the material properties in nanoindentation [35,36]. In order to study the influence of defective twin boundary on the contact hardness of nt-Cu, the hardness-contact strain responses are plotted in Figure 3. The contact hardness in nanoindentation is defined as in [37,38]

(4) $$H=\frac{P}{A_{p}}$$

where P is the indenter force, and $A_{p}$ is the projected contact area. There are several ways for calculating the projected contact area. In this work, we have chosen an elliptic approximation of projected contact area which is more reliable in relatively large nanoindentation [39]:

(5) $$A_{p}=\frac{\pi }{4}\left(x^{\max}-x^{\min}\right)\left(y^{\max}-y^{\min}\right)$$

where the $x^{\max}$ and $x^{\min}$ are the maximum and minimum x coordinates of the atoms that are in contact with the indenter, so does y dimension. Noting that although the contact area is calculated in this way, in order to obtain the contact radius, the edge of the contact area is still treated as an approximate circle. Thus, the contact radius a could be calculated as

(6) $$a=\sqrt{\frac{A_{p}}{\pi }}$$

The non-dimensional contact strain in spherical indentation could be expressed as a/R.

As shown in Figure 3, the hardness of three different models increases linearly with the increase of contact strain in the elastic stage. Noting that the contact strain during the indentation process does not increases monotonically with the increase of the indenter depth, because the radius of the contact area in molecular dynamic simulation does not always increase even in the elastic stage. These hardness data are in good agreement with some simulation results [33,39,40]. The linear fit of hardness in elastic stage are also shown in dash line in Figure 3, and the R-squares of three various models all exceed 0.99 as listed in Table 1. As indenters are continuously pushed into the material until the plastic stage, the hardness of material comes to a critical value then suddenly decreases a lot. This sharp decline is caused by the formation of the incipient plastic structure. The nucleation of the first dislocation beneath the indenter not only decreases the force on the indenter but also increases the contact area between the indenter and the surface, which cause the decline of hardness. In addition, the maximum hardness of three various models are also presented in Table 1. It can be seen that the critical hardness in SDT and TDT are smaller than the critical hardness in TPT. The inherent kink-like defects in twin boundary will cause the decrease of the maximum critical hardness of nanotwinned copper. This is due to the differences between the incipient plastic structures in three different models which will be discussed below in detail.

### 3.3. Surface Accumulation Analysis

The surface morphology is an important feature of thin films, which could affect the mechanical properties and performance of materials. The accumulation of surface atoms in nanotwinned copper will significantly change the surface morphology of materials. So it is valuable to investigate the change of surface accumulation situation around the indenter of materials under highly localized stress.

**Figure 4:**
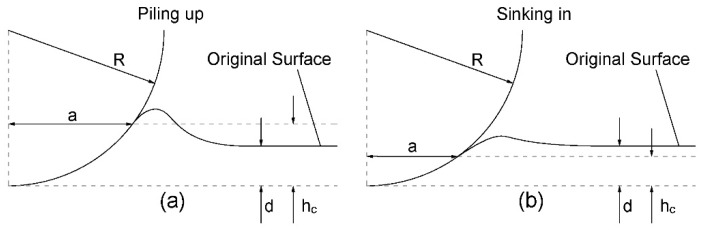
Schematic representation of surface morphology during spherical nanoindentation process. (**a**) “Piling up” model and (**b**) “Sinking in” model.

During the spherical nanoindentation, the circle delimiting contact between the indenter and the material usually is not at the same plane of the original surface, but maybe above or below it [36,41]. Hence, atoms near the indenter may be stacked up and surround the indenter, or could be pushed away on the surface from the indenter or pushed deeply into the material. As illustrated in Figure 4a, if the height of contact circle is higher than the original surface, the nanoindentation process at that moment is “piling up” model; while the contact circle is lower than the surface, the process at that moment is “sinking in” model as shown in Figure 4b. In Figure 4, *d* and *h_c_* are the indenter depth and the contact depth, respectively. The *h_c_* can be obtained by the equation below

(7) $$h_{c}=R-\sqrt{R^{2}-\frac{A_{p}}{\pi }}$$

Here *h_c_* is an average value of the contact depth of the indenter, because *A_p_* is an approximate average. If *d* < *h_c_* (i.e., piling up model), many atoms will stack on the surface around the indenter, and the number of these stacked atoms increases with the increase of the contact radius. Else if *d* > *h_c_* (sinking in model), the surface of the material is relatively flat.

This atom accumulation phenomena related to “piling up” and “sinking in” will directly affect the surface morphology of the material around the indenter which is important in engineering applications. In addition, this “piling up” or “sinking in” results from the distortion of the surface by material displaced by indentation, and it is determined by the strain hardening properties of the material [41]. So it is helpful for understanding the strain hardening behavior by analyzing the change of this model of surface contact situation.

**Figure 5:**
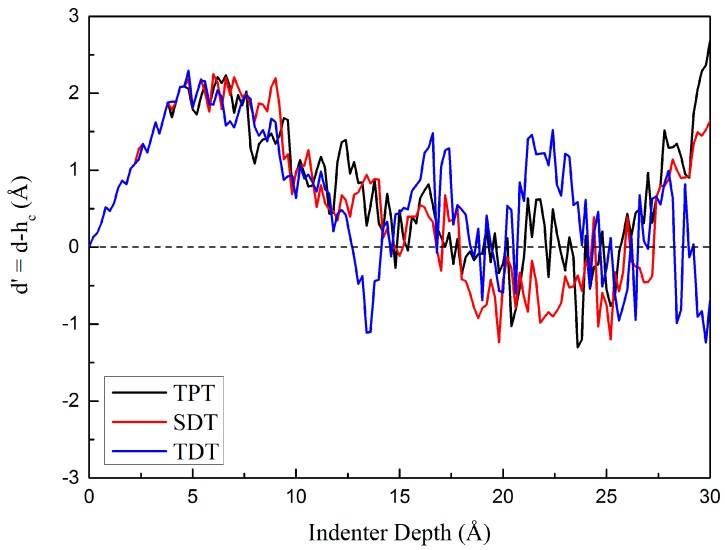
Curves of d′ with the indenter depth of three different models.

In order to quantitatively evaluate the roughness and accumulation degree of the part of surface around the indenter, we firstly define $d^{\prime }=d-h_{c}$ to quantitatively evaluate the changes of the surface contact model. Here *d* and *h_c_* are the indenter depth and the contact depth, respectively, and *d*′ is an average value of the accumulation situation around the indenter. The changes of *d*′ with the increase of indenter depth of three various models are shown in Figure 5.

Results show that *d*′ increases with the increase of indenter depth at the elastic stage, so the indenter is sinking in the material and there is no atom accumulation during the initial elastic indentation. As the indentation proceeds, *d*′ begins to decrease when the first dislocation nucleates beneath the indenter. At the plastic stage, *d*′ goes up and down frequently due to the abundant changes of the inner structure. When *d*′ decreases, that means the contact depth increases faster than the indenter depth and the number of piling up atoms on the surface increases. That will lead to the change of the surface morphology. By comparing the *d*′ curves of three various models as shown in Figure 5, it can be inferred that with the increase of the amount of the kink-like defects in twin boundaries, the surface morphology around the indenter changes more and more frequently. Which means that the kink-like defects could enhance dislocation reactions, particularly in TDT. We can conclude that the interaction between the defects in two twin boundaries in TDT will apparently promote the dislocation nucleation and interactions. This will be discussed quantitatively below.

### 3.4. Incipient Plastic Structures

**Figure 6:**
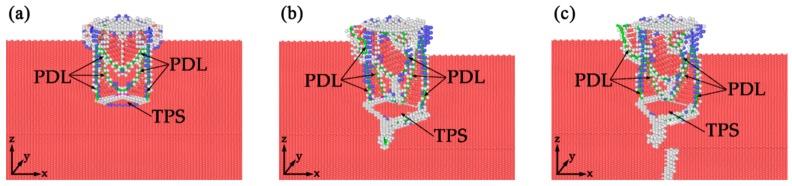
Initial defect structures in TPT, SDT, and TDT, respectively. The indenter depth is d = 5.0 Å. (**a**) TPT; (**b**) SDT; (**c**) TDT.

We presented the initial defect structures in TPT, SDT, and TDT after yielding event in Figure 6, and the indenter depth for these figures is *d* = 5.0 Å. Note that we use the dislocation extraction algorithm (DXA) [30] and common neighbor analysis to identify the dislocations in the plastic zone and the types of the local lattice structures. The color of atom represents the local lattice structure of the atom, where green for FCC, red for HCP, blue for BCC, and gray for “other” lattice structures. The atoms on the twin boundaries are regarded as HCP atoms, and atoms on the defects at the twin boundaries are assigned as “other” type atoms. Additionally, atoms in the upper and bottom surfaces of the bulk system are assigned as “other” type too. In order to clearly reveal the dislocation structures under the surface, we hide the FCC atoms and surface atoms. The colored lines in the structures labeled in the figures represent dislocation lines.

It is clear that the defect structure in TPT is different from the other two models as shown in Figure 6a. The incipient plastic structure in TPT is a symmetric structure, Shockley partials marked in figures (Shockley partial dislocation line, PDL) are emitted from the indented surface, following with stacking faults. When the leading partials reach the twin boundaries, twin boundary migration occurs and twinning partial slip [42] (TPS) forms on the upper twin boundaries. Part of the dislocation is absorbed by the twin boundary, which introduces dislocations in the upper twin boundary plane. While the dislocation structures beneath the indenters in SDT and TDT are asymmetrical and more complicated, as shown in Figure 6b,c. The twin boundaries migration also happen and the twinning partial slip also form on the twin boundaries. The leading partials in SDT and TDT interact with the intrinsic defects when the dislocations reach on the twin boundary, but not interact with twin boundary directly. This cause the difference of twinning partial slip on the twin boundary, which will be discussed in next section. It can be concluded that the yielding event corresponds to the nucleation and emission of the dislocations beneath the indenter, and the changes of critical loads and hardness are caused by the absorption of dislocation by the twin boundary. This conclusion is in good agreement with some in-situ investigations [19,34].

### 3.5. Twinning Partial Slip

#### 3.5.1. Formation of Twinning Partial Slips

The twinning partial slip caused by the twin boundary migration is an important mechanism in nanoindentation. The formation and changes of the twinning partial slips in three different models will be discussed in this part.

**Figure 7:**
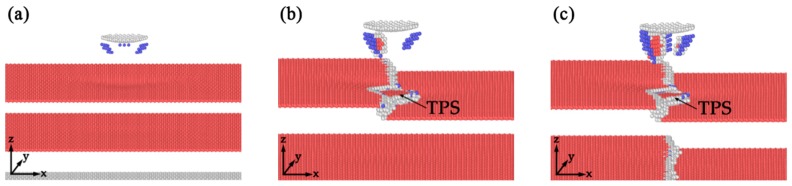
Atomistic configurations of three different models at indenter depth d = 4.8 Å. (**a**) TPT; (**b**) SDT; (**c**) TDT.

One notable phenomenon during the formation of incipient plastic structure is that, the twinning partial slip on the twin boundary appears before the leading partials reach the first twin boundary in SDT and TDT. The atomic configurations of three different models before yielding point are shown in Figure 7. As shown in Figure 7a, the twinning partial slip forms until the leading partial reaches the twin boundary in TPT. Figure 7b,c show that the formation of twinning partial slips already happens before the dislocations contact the defects in SDT and TDT. As the indenter moves downward, the stress on the upper twin boundary gradually concentrates on the location where twinning partial slip is generated. Then the inherent partial dislocations in the kink-like defects become unstable and dislocation reaction happens. The stress for activating the dislocation reaction of the intrinsic dislocation in the twin boundary is smaller than that needed for twin migration on perfect twin boundary, such that the twinning partial slips of the incipient plastic structures in SDT and TDT generate earlier than the generation of twinning partial slip in TPT. The phenomenon also show that the kink-like defects could introduce instability to the twin boundary.

#### 3.5.2. Twinning Partial Slips in Incipient Plastic Structures

**Figure 8:**
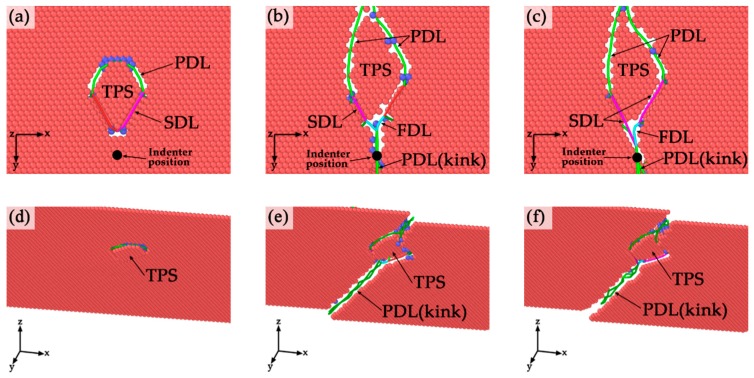
Twinning partial slips on the upper twin boundaries in TPT, SDT, and TDT in bottom view and bottom side views. The indenter depth of these figures is d = 5.0 Å. (**a**–**c**) Bottom view in TPT, SDT, and TDT; (**d**–**f**) Bottom side view in TPT, SDT, and TDT.

In order to compare the differences between the twinning partial slips of incipient plastic structures of three different models, the bottom views of the upper twin boundaries are shown in Figure 8a–c. The projection positions of the indenters are marked as black points in the figures to clearly reveal the relative positions of twinning partial slips. It is shown that the twinning partial slips are not located directly below the indenter, because the dislocations emission direction ([112] in our simulation) is inclined with twin boundary and the angle between PDL and twin boundary is about 70°. In Figure 8a, the twining partial slip in TPT is surrounded by partial dislocation lines (PDL) and stair-rod dislocation line (SDL). However, in SDT and TDT, in addition to Shockley partial dislocation lines and stair-rod dislocation line, Frank dislocation line (FDL) is also detected on the twin boundary as shown in Figure 8b,c. Note that the differences between the configurations in Figure 8b,c of twinning partial slips in SDT and TDT is very small, because the indentation is still very shallow at this stage and the lower twin boundary could not affect the upper plastic structure. In addition, it can be observed that the existence of kink-like defect could expand the area of twinning partial slip on the twin boundary in incipient plastic structure, and this will also cause the decrease of the critical hardness. From the observation of the bottom side view of the upper twin boundaries of three various models (Figure 8d–f), we can clearly see that the kink-like defects in twin boundary are regarded as partial dislocations. The twinning partial slips in SDT and TDT are the results of the interactions between the leading partial emitted under the indenter and the inherent partial dislocation at the kink defects.

From the analysis of Figure 8, it can be inferred that the kink-like defect could expand the area of twinning partial slip on the twin boundary. In addition, as shown in Table 1, the critical hardness in SDT and TDT is smaller than the critical hardness in TPT. Thus, it can be concluded that the twin boundary migration and the formation of twinning partial slip will decrease the hardness of material. This phenomenon is also detected in results of experiments [19]. Furthermore, the interaction between kink-like defects and leading partials could introduce Frank dislocation to the twin boundary, which could not nucleate on perfect twin boundary during interaction between leading partial and twin boundary itself.

#### 3.5.3. Expansion of Twinning Partial Slips

**Figure 9:**
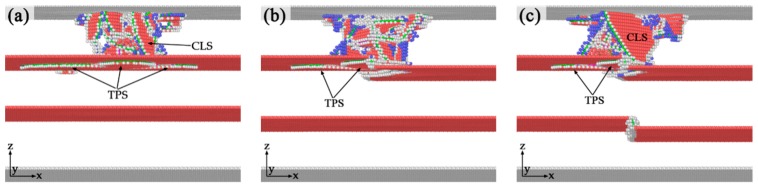
Atomic configurations of dislocation structures of three different models at indenter depths d = 9 Å. (**a**) TPT; (**b**) SDT; (**c**) TDT.

In order to investigate the development of twinning partial slips on the upper twin boundaries, the atomic configurations of dislocation structures in TPT, SDT, and TDT at indenter depth d = 9 Å are shown in Figure 9. Note that at this stage, the lower twin boundaries in SDT and TDT have no effect on the expansion of the twinning partial slip in the upper twin boundary. It is clear that with the proceeding of the indentation after the formation of incipient plastic structures, the areas of twinning partial slips on the upper twin boundaries in all three various models are gradually extending. Simultaneously, some confined layer slips (CLS, the slip plane of dislocation is inclined to twin boundary, but the Burgers vector is parallel with twin boundary) [42] are detected in the top grain, due to the dislocation emission under the indenter. As indenters were pushed more deeply, more partial dislocations nucleate and move downward. These dislocations accumulated on the twin boundaries, and some of these dislocations were absorbed by the twin boundaries. This phenomenon is in good agreement with experiments [19,43]. The twin boundary migration phenomenon becomes intense, which results in the expansion of twinning partial slips under the upper twin boundaries. As shown in Figure 9a, the twinning partial slip expands in both left and right directions in TPT. The twinning partial slips in models with defective twin boundaries (SDT, TDT) only expand in half plane of the twin boundary which is relatively higher than another half in z dimension, as shown in Figure 9b,c. This means that the kink-like defect could block the extension of twinning partial slip in the twin boundary.

### 3.6. Dislocation Transmission

The incipient plastic structures and the twinning partial slips have been discussed in previous sections. Furthermore, the dislocation transmission is another important mechanisms between dislocations and twin boundaries. Here we analyze the dislocation transmissions in TPT, SDT, and TDT to discuss the effect of kink-like defects in twin boundaries on the dislocation transmission.

**Figure 10:**
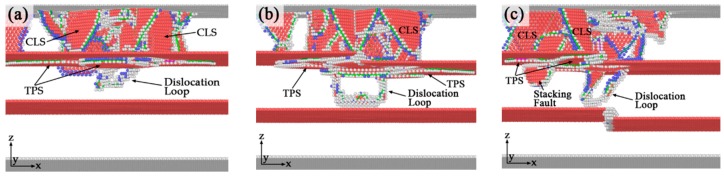
The atomistic configurations of dislocation transmissions of three different models. (**a**) TPT, d = 14.4 Å; (**b**) SDT, d = 16 Å; (**c**) TDT, d = 13.6 Å.

Figure 10 shows the atomic configurations of dislocation transmissions across the upper twin boundary in three different models. With indentation process proceeding, many twinning partial slips form under the upper twin boundaries. The slip along the twin boundaries is increased due to the preexisting defects which act as the origins of partial dislocation emissions, as in agreement with previous observations that defects is an important nucleation site of twinning partials [16]. The multiplication of twinning dislocation at the twin boundary under indentation is also confirmed by previous experiments where it is related with a variation of the magnitude and orientation of the Burges vector of the twin boundary defects [44]. It can be seen in Figure 10a that in TPT, the plastic structure crosses through the upper twin boundary when indenter depth comes to 14.4 Å. A dislocation loop nucleates under the upper twin boundary in the middle grain, and it is emitted toward the lower twin boundary. While in SDT (Figure 9b), the dislocation transmission across the upper twin boundary happens until d = 16 Å. The pattern of twin boundary migration and transmission across twin boundary event in our simulation is also investigated in some experiments [19,34,43]. Fang et al. [17] found that in tensile simulation, a single kinked defective twin boundary could change the mechanisms from direct dislocation transmission to dislocation absorption, which is in good agreement with our simulation result. So it is reasonable to explain that the delay effect of the dislocation transmission is caused by the interaction between the dislocation and kink-like defect. It is also noticed that dislocation transmission is almost the only source of interaction between twin boundaries. However, dislocation transmission happens at d = 13.6 Å in TDT (Figure 9c), which is earlier than the time of transmission in TPT and SDT. It can be inferred that two adjacent defective twin boundaries will promote the twin boundary interactions and advance the dislocation transmission process.

**Figure 11:**
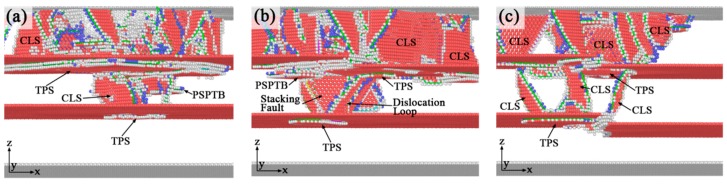
The formation of twinning partial slips on the lower twin boundaries in three different models. (**a**) TPT, d = 26.8 Å; (**b**) SDT, d = 22.4 Å; (**c**) TDT, d = 16.6 Å.

Figure 11 shows the formation of twinning partial slips on the lower twin boundaries of three different models. As indentation continuous, the side segment (inclined to twin boundary) of the full dislocation loop transforms into extended dislocation segment (two Shockley partials with a stacking fault between them). In addition, some partial slips that are parallel with twin boundary (PSPTB [42], slip plane and Burgers vector are both parallel with twin boundary) are also detected. As indenter is pushed more deeply, the dislocations will contact with the lower twin boundary and also cause the generation of twinning partial slip on the lower twin boundary. For TPT, the twinning partial slip forms on the lower twin boundary when indenter depth equals to 26.8 Å (Figure 11a). For SDT and TDT, the corresponding indenter depths are 22.4 Å and 16.6 Å, respectively (Figure 11b,c). The reason of the substantial advance of this dislocation contact on the lower twin boundary in TDT is that the interactions between the defects in upper and lower twin boundaries promote the dislocation emission process. It can be seen that, the dislocation structure in materials becomes more and more complicated with the increase of the kink-like defects in the twin boundaries. Compared with STD, more confined layer slips form across the grain, and more twinning partial slips generate under the twin boundaries in TDT. Due to the above analysis, it can be concluded that one single defective twin boundary could delay the dislocation transmission across the twin boundary, while two adjacent defective twin boundaries will advance the dislocation emission process, accelerate the dislocations nucleation and emission, and also will enhance the interactions between twin boundaries.

### 3.7. Dislocation Length Quantitative Analysis

The atomic configurations of dislocation structures have been shown in previous section, and influence of the kink-like defect of twin boundary on deformation mechanisms during nanoindentation have also been discussed. In order to further understand the change of the plastic structures, dislocation extraction algorithm (DXA) [29] are employed in this section to quantitatively evaluate the dislocation length during the indentation process.

**Figure 12:**
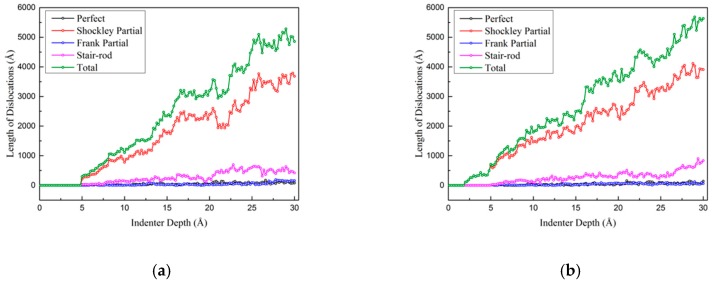

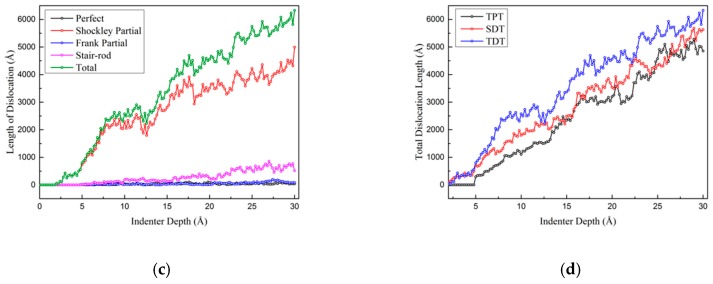
Dislocation length change of three different models. (**a**) TPT; (**b**) SDT; (**c**) TDT; (**d**) Total dislocation lengths of three models.

Figure 12a–c shows the changes of the lengths of different kinds of dislocations during nanoindentations of the three models. It is clear that most nucleated and emitted dislocation is Shockley partial in all three models, which means that the plastic zone develops by the nucleation and emission of Shockley partials. It is worth mentioning that the nucleations of dislocations in SDT and TDT happen earlier than the nucleation in TPT, as shown in Figure 9b,c. The reason of this phenomenon is that the kink-like defect itself is a core of dislocation nucleation, and the intrinsic existence of defects makes the defective twin boundary relatively unstable compared to perfect twin boundary. When indenter has been pushed at a critical depth, the stress concentrated on the defect becomes big enough to nucleate dislocation directly on the defects. While in TPT model, no dislocation will be nucleated at the same indenter depth due to the stability of the perfect twin boundary. The stress needed for dislocation nucleation on defective twin boundary could not cause the nucleation phenomenon on perfect twin boundary.

Figure 12d shows the total dislocation length of three different models during deformation process. It can be seen that dislocation length in TDT is more than other two models, and the dislocation length of SDT is longer than the length in TPT for most of time during indentation process. Therefore, it can be concluded that the existence of kink-like defects in twin boundary will promote the nucleation of dislocation and dislocation interaction during nanoindentation. The dislocation length of the structures increases with the increase of the amount of kink-defects in the twin boundaries. In addition, interaction between the defects in two adjacent twin boundaries will cause abundant interactions between the twin boundaries.

## 4. Conclusions

Effects of kink-like defect in twin boundary on deformation mechanisms and interactions between dislocations and kink-like defects in nanotwinned Copper thin film during nanoindentation was studied by molecular mechanics. Nanotwinned Copper (nt-Cu) with two defective twin (TDT) boundaries was compared with nt-Cu with two perfect twin (TPT) boundaries and nt-Cu with one perfect twin (SDT) boundary and one defective twin boundary. Force-indenter depth responses and hardness-contact strain curves were obtained. Results show that defects in the twin boundary will reduce the critical indenter force and the critical hardness of the material. This is in good agreement with some experiments.

A quantitative indicator was proposed to evaluate the surface atom accumulation during nanoindentation. Results show that the intrinsic defects could affect the surface atom accumulation around the indenter. The surface morphology in TDT changes more frequent than surface in TPT and surface in SDT, which means two defective twin boundaries could significantly enhance the dislocation reactions.

The atomistic configurations of incipient plastic structures were investigated. The phenomenon of accumulation of dislocation and pattern of twin boundary migration in our simulation is in agreement with experiments. It is found that the intrinsic existence of kinks in twin boundary will change the incipient plastic structure, and Frank partial was detected on the upper twin boundaries in SDT and TDT which was not found in TPT. The development of twinning partial slips of three different models were discussed. Twinning partial slip forms before the dislocation reaches the twin boundary in SDT and in TDT, while twinning partial slip forms until the dislocation contact the twin boundary in TPT. The areas of the twinning partial slip in incipient plastic structures in SDT and in TDT are both larger than the twinning partial slip area in TPT, which cause the decrease of the critical load and critical hardness of material. In addition, the intrinsic kink-like defects could block the extension of the twinning partial slip in the twin boundary.

The processes of dislocation transmission were analyzed by discussing the atomistic configuration. Results reveal that the dislocation transmission could be delayed in model containing single defective twin boundary. The transmission process could be advanced in model containing two consecutive defective twin boundaries with same defect position, and the plastic deformation could be enriched. The transmission pattern in our simulation is in agreement with some experiments results.

Furthermore, the evolution of lengths of different kinds of dislocations of three various models during deformation processes were quantitatively analyzed. It can be seen that the defects in the twin boundary could advance the nucleation of new dislocations, and two consecutive twin boundaries could promote the plastic deformation. It is shown that the most nucleated dislocation during indentation is Shockley partial, and defect in twin boundary could promote the nucleation of dislocations, dislocations interaction and twin boundaries interaction.

Therefore, in some cases, it is recommended to produce a single defective twin boundary in the nanotwinned thin film structure to delay the dislocation transmission event and block the twin boundary migration. On another hand, these findings are also helpful to understand the mechanisms of interaction between dislocations and intrinsic defects on twin boundary in “plain” materials like nanotwinned copper. Our study shows that the inherent kink-like defects play a crucial role in the deformation mechanisms and it should be taken into consideration in future researches on the nanotwinned materials.

## Figures and Tables

**Table 1:** Adjust R-Squares of three linear fits and critical hardness of three models.

Model	Adjust R-Square of Linear Fit	Maximum Hardness (GPa)
TPT	0.99087	27.74
SDT	0.99121	26.98
TDT	0.99163	27.11
